# Efficient antioxidant defence systems of spring barley in response to stress induced jointly by the cyst nematode parasitism and cadmium exposure

**DOI:** 10.1007/s11104-020-04713-y

**Published:** 2020-09-14

**Authors:** Mateusz Labudda, Ewa Muszyńska, Marta Gietler, Elżbieta Różańska, Anna Rybarczyk-Płońska, Justyna Fidler, Beata Prabucka, Abdelfattah A. Dababat

**Affiliations:** 1 Department of Biochemistry and Microbiology, Institute of Biology, Warsaw University of Life Sciences-SGGW, Nowoursynowska 159, 02-776 Warsaw, Poland; 2 Department of Botany, Institute of Biology, Warsaw University of Life Sciences-SGGW, Warsaw, Poland; 3 International Maize and Wheat Improvement Center (CIMMYT), Soil Borne Pathogens Program, Ankara, Turkey

**Keywords:** Antioxidants, Cross-talk between *Heterodera* filipjevi and cadmium, *Heterodera filipjevi*, *Hordeum vulgare*, Oxidative stress, Trace metal

## Abstract

**Aims:**

This research aimed to establish how *Hordeum vulgare* responds to abiotic and biotic stress affecting in tandem.

**Methods:**

Plants were inoculated with *Heterodera filipjevi* and treated with cadmium (Cd) concentration (5 μM) that can occur in the cultivated soil. To verify the hypothesis about participation of increased antioxidative defence in *H. vulgare* under stress, biochemical and microscopic methods were implemented.

**Results:**

The amount of superoxide anions and hydrogen peroxide was diminished in plants that were both nematode-inoculated and cadmium-treated. Superoxide anions were rendered harmless by increased activity of superoxide dismutase, and H_2_O_2_ was scavenged via Foyer-Halliwell-Asada pathway. The unique enhanced antioxidant capacity of double stressed plants was also linked with the accumulation of *S*-nitrosoglutathione as nitrosoglutathione reductase activity was inhibited. Furthermore, stimulated activity of arginase in these plants could promote polyamine synthesis and indirectly enhance non-enzymatic antioxidant mechanism. Results indicate that different antioxidants operating together significantly restricted oxidation of lipids and proteins, thus the integrity of cell membranes and protein functions were maintained.

**Conclusions:**

The ROS deactivation machinery in barley leaves showed an unusual response during stress induced by *H*. *filipjevi* infection and cadmium treatment. Plants could induce a multi-component model of stress response, to detoxify Cd ions and efficiently repair stress damage.

## Introduction

Barley (*Hordeum vulgare* L.) is a globally important cereal crop. Its grains are used to produce human food, livestock feed and beverages by malting, brewing, and distilling industries as well as biofuels (Tricase et al. 2018). In natural conditions, crop plants, including spring and winter barley are affected by a plethora of environmental stresses. Particularly in the face of global climate changes, which are becoming more and more noticeable, it is rarely seen that crop plants need to deal with only one environmental stress during their growth and development. A much more common case is the situation when plants have to response to several abiotic or biotic stresses operating simultaneously or sequentially. Plants draw the mineral nutrients from the soil for building organic compounds and finally for yielding. Moreover, soil is a source of many stress factors (Raza et al. 2019).

Among the soil origin abiotic stresses are water deficit or its excess, salinity, nutrient imbalance, unfavourable pH (Niedziela et al. 2014; Pandey et al. 2017). What is more, soils in many areas around the world are strongly polluted with trace metals and metalloids, including cadmium (Cd) as a non-essential highly toxic element (Muszyńska et al. 2019c). The Cd pollution in agricultural regions is mostly due to the application of contaminated sewage sludges, phosphate fertilizers, irrigation water and plant protection products (Paunov et al. 2018). Cd can accumulate in the grains of cereal plants and then can cause serious problems with health of the livestock and human and with the crop production (Labudda 2011; Deng et al. 2019). Cd also significantly affects plant growth and development as well as physiological processes including among others photosynthesis, nutrient metabolism, and antioxidant machinery (Gill and Tuteja 2010). Besides abiotic stressors, the most economically significant groups of pathogens such as cyst nematodes of the genus *Heterodera* and *Globodera* and viruses (wheat mosaic virus and barley yellow mosaic virus), protist *Plasmodiophora brassicae*, and fungus and fungus-like species of the genus *Phytophthora*, *Pythium*, *Rhizoctonia*, *Fusarium*, and *Verticillium* are soil-borne pathogens of plants (Labudda 2018). Cereal cyst nematodes are closely related parasitic species that attack cereals. Until now, eleven cereal cyst nematodes species have been described. Of these *Heterodera latipons, H. avenae* and *H. filipjevi* are the most economically important pathogens for the worldwide production of cereals (Toumi et al. 2018). The rye cyst nematode *H. filipjevi* (Madzhidov) Stelter is an obligate biotrophic sedentary parasite, mainly infecting barley and wheat (Dababat and Fourie 2018). The *H. filipjevi* parasitism on roots of cereal plants causes the grain yield losses reach up to 50% (Pariyar et al. 2016).

In view of the classical Levitt’s theory (Levitt 1972), which shows the plant physiological mechanisms during response to stress, different abiotic and biotic stressors may be considered as primary stress and then as its result, a secondary stress may appear. In plant organisms, the secondary stress is always linked to the changes of redox balance in cells, so oxidative stress may be induced. Oxidative stress is defined as an imbalance between the production of reactive oxygen species (ROS) and an effectiveness of the cellular systems to neutralize them. ROS such as singlet oxygen, superoxide anion, hydroxyl radical or hydrogen peroxide are continuously produced during various kinds of metabolic processes, including photosynthesis and respiration. The ROS increased content in plants can lead to damage of nucleic acids, proteins, lipids, carbohydrates, and plant pigments (Muszyńska and Labudda 2019; Muszyńska et al. 2019a). To preclude the impoverishment of cellular functions, the ROS level is controlled by enzymatic and non-enzymatic antioxidant systems. The enzymatic antioxidant system encompasses mainly superoxide dismutase (SOD), catalase (CAT), various peroxidases and enzymes of the ascorbate-glutathione pathway (Labudda and Azam 2014; Muszyńska et al. 2018b; Kapoor et al. 2019), whereas non-enzymatic system consists of reduced molecules of glutathione (GSH) and ascorbate (ASA) and a large group of phenolic metabolites (Saxena et al. 2016; Durak et al. 2019; Muszyńska et al. 2020). Salicylic acid (SA) is described as a phenolic phytohormonal player during signalling and regulating of the numerous plant responses to abiotic and biotic stresses (Morkunas et al. 2011, 2018; Maruri-López et al. 2019).

In published literature, many research results demonstrate plant responses to Cd (for review see Martinka et al. (2014) and Loix et al. (2017)). However, the applied Cd concentrations were often not realistic and significantly differ from the Cd level in contaminated soil (Sanità di Toppi and Gabbrielli 1999). Therefore, based on published results by Milone et al. (2003) and Muszyńska et al. (2018a), we chose a realistic concentration of Cd which can occur in the cultivated soil. Therefore, we hypothesized that the enhanced antioxidative defence system could play a role of a great importance in the plant systemic physiological mechanisms to resist oxidative stress and damage induced dually by the cyst nematode parasitism and Cd exposure. To find that out, we used a biochemical-physiological approach in combination with microscopic observations. This paper highlights how spring barley plants respond to dual environmental stresses, namely the cyst nematode infection and Cd exposition.

## Materials and methods

### Plants and growth conditions

Seeds of the spring barley *Hordeum vulgare* L. cv. ‘Airway’ were washed in tap water for 2 h and next they were surface decontaminated in 5% NaOCl with 0.2% Tween 20 for 10 min with stirring. They were rinsed under tap water for 1 h and next incubated for 1 h in 0.2% Plant Preservative Mixture (PPM) (Plant Cell Technologies, Inc., Washington DC, USA) to exclude potential contamination by microorganisms. The decontaminated barley seeds were put (embryos upwards) side by side into Petri dishes (9 cm diameter) on a 0.2% PPM-soaked filter paper and covered. After 18 h incubation at 4 °C in the dark, the seeds were kept in the dark at 23 °C for 2 days (Labudda et al. 2020b). Twelve germinated seeds were planted into a plastic pot (25 × 25/26 cm) with saucer. Pot was filled with a commercial horticultural substrate consisted of a mixture of low-moor and high-moor peats (0–20 mm fraction). Substrate had no addition of mineral fertilizers, its pH in water was in the range of 5.6–6.8 and before planting it was autoclaved at 121 °C, 0.1 MPa for 20 min. To each pot aliquot of one hundred and fifty ml of sterile 0.2 × Knop medium (pH 6.4) was added. Barley plants were cultivated in a growth chamber MLR-350 (Sanyo, Tokyo, Japan) at 25 °C during the day and at 23 °C at night with a 16 h/8 h day/night cycle under a photosynthetic photon flux density of 100 ± 25 μmol/m^2^/s and at 50% humidity. Every 2 days plants were watered with 100 ml sterile milli-Q water.

### Cyst nematode inoculum preparation

Cysts of *Heterodera filipjevi* (Madzhidov) Stelter were collected from naturally cyst nematode-settled experimental wheat fields of the International Maize and Wheat Improvement Center (CIMMYT) in Yozgat (39°08’N, 34°10′E; altitude 985 m a.s.l.) in the Central Anatolian Plateau of Turkey. Cysts were extracted from rhizospheres and roots of *Triticum aestivum* plants harvested at the end of the growing season. The modified extraction protocol presented by Ashrafi et al. (2017) was used. Hatching of pre-parasitic *H. filipjevi* juveniles was provoked by the cyst incubation in sterile 3000 μM zinc chloride at 17 °C. Freshly hatched pre-parasitic juveniles were carefully washed 6 times in sterile milli-Q water and then were suspended in water.

### Plant inoculation and cadmium treatment

Pots with 7-day-old plants were divided into four groups, nematode-uninoculated and cadmium-untreated controls (C), nematode-inoculated (N), cadmium-treated (Cd) and both nematode-inoculated and cadmium-treated (N + Cd) plants. Plants from the N and N + Cd groups were inoculated with approximately 2400 freshly hatched pre-parasitic *H*. *filipjevi* juveniles suspended in sterile milli-Q water per pot and plants were watered with 100 ml sterile milli-Q water. Plants from the Cd and N + Cd groups were watered with 100 ml sterile milli-Q water enriched with 5 μM CdCl_2_. C plants were watered as well. Barley plants from all these experimental groups were collected after 14 days of stress exposition. This short-term experimental time was chosen based on our previous published observations (Labudda et al. 2020b) reflected the dynamics of growth and development of *H*. *filipjevi* larvae in spring barley (the same cultivar as tested in this article) roots under conditions of pot experiment. At 14 days post-inoculation (dpi), most sedentary J2 larvae moulted to J3 larvae, which indicated that the 14 dpi syncytia met their nutritional demands. About 14 dpi, the J3 larvae fed intensively to reach the J4 stage and sexual maturity after 21 dpi and start reproducing. Four plants were used for each bulked sample and experiments were conducted in three biological replicates.

### Evaluation of physiological status and antioxidative system of barley plants

#### Photosynthetic pigments

The amounts of photosynthetic pigments were assayed according to Lichtenthaler (1987). Leaf samples (100 mg) were macerated in 10 ml of ice-cold 80% acetone with addition of calcium carbonate and centrifuged for 15 min at 4 °C (16,000×g). The absorbance of acetone extracts was measured at 470, 646 and 663 nm in Nunc U-bottom 96-well plate (Thermo Scientific, Waltham, MA, USA) on a Varioskan LUX Multimode Microplate Reader (Thermo Scientific, Waltham, MA, USA). The chlorophyll *a* (chl *a*), chlorophyll *b* (chl *b*) and carotenoid amounts were calculated according to Wellburn (1994). Total chlorophylls (chl *a* + *b*), the ratio of chlorophyll *a* to *b* (chl *a*:*b*) and the ratio of chl *a* + *b* to carotenoids (chl *a* + *b*:carotenoids) were also estimated.

#### Superoxide anions and hydrogen peroxide (H_2_O_2_)

Measurement of superoxide anion contents was performed according to Doke’s method (Mai et al. 2013). Barley leaves (100 mg) were immersed in 1 ml of the 0.01 M K/Na phosphate buffer (pH 7.8) containing 0.05% nitro blue tetrazolium (NBT) and 0.01 M sodium azide. Samples were incubated in the dark for 1 h at room temperature. After incubation, reaction solutions (without leaves) were heated at 85 °C for 15 min and rapidly cooled on ice-bath. Superoxide anions content was expressed as absorbance at 580 nm per gram of fresh weight (FW).

The H_2_O_2_ content was estimated according to procedure by Junglee et al. (2014). Leaf samples (100 mg) were homogenized in mixture containing 0.25 ml of 10 mM K/Na-phosphate buffer (pH 5.8), 0.25 ml of 0.1% trichloroacetic acid and 0.5 ml of 1 M KI. Supernatants obtained by the leaf homogenates centrifugation (4 °C, 15 min, 16,000×g) were kept in the dark for 20 min at room temperature. Next, the samples were centrifuged (10 min, 16,000×g), and the absorbance was measured at 350 nm in Nunc U-bottom 96-well plate on a Varioskan LUX Multimode Microplate Reader. The H_2_O_2_ content was estimated from a standard curve and calculated per gram of FW.

#### Enzymatic parameters

Leaf samples (200 mg) of barley plants were homogenized in a mortar with quartz sand and 2 ml of ice-cold extraction buffer (pH 7.2) containing 50 mM tris(hydroxymethyl)aminomethane (Tris)-HCl, 2 mM 2-mercaptoethanol, 1 mM ethylenediaminetetraacetic acid (EDTA), 5% glycerol, 1 mM phenylmethylsulfonyl fluoride, 5 mM MgCl_2_, 2% polyvinylpyrrolidone. Homogenates were centrifuged (4 °C, 20 min, 16,000×g) and supernatants were collected.

Superoxide dismutase (SOD) activity was measured according to Kostyuk and Potapovich (1989). A reaction reagent was prepared by mixing equal volumes of 0.067 M K/Na phosphate buffer (pH 7.8) and 0.025 M EDTA. The pH value of this reagent was adjusted to 10.0 by tetramethylethylenediamine. Next, 0.05 ml of reaction reagent and 0.12 ml of milli-Q water were added to 0.005 ml of supernatant. The enzyme reaction was started by the pipetting of 0.005 ml of 2.5 μM quercetin in dimethyl sulfoxide. Measurements were conducted in Nunc U-bottom 96-well plate on a Varioskan LUX Multimode Microplate Reader. The absorbance at 406 nm was measured for 20 min with reads every 1 min. The one arbitrary unit of SOD activity was defined as 0.01 decrease of absorbance after 1 min per gram of FW.

Catalase (CAT) activity was measured according to Aebi (1984). The supernatant (0.002 ml) was mixed with 0.018 ml of 0.05 M Tris-HCl buffer (pH 7.2) and 0.01 ml of 0.168% hydrogen peroxide in the same buffer. Measurements were conducted at 37 °C in UV-Star 96-well plate (Greiner, Monroe, NC, USA) on a Varioskan LUX Multimode Microplate Reader. The absorbance at 240 nm was recorded for 10 min with reads every 1 min. The CAT activity was expressed as a decomposition of μmol of hydrogen peroxide per minute and gram of FW.

Peroxidase activity (POD) was measured according to Lück (1962). The supernatant (0.005 ml) was mixed with an activity reagent consisting of 0.49% p-phenylenediamine and 0.049% hydrogen peroxide in 0.05 M Tris-HCl buffer, pH 7.2 or 8.8). Measurements were performed at 37 °C in Nunc U-bottom 96-well plate on a Varioskan LUX Multimode Microplate Reader. The absorbance at 485 nm was recorded for 10 min with reads every 1 min. The POD activity was expressed in arbitrary unit, separately for pH 7.2 (POD_7.2_) and 8.8 (POD_8.8_). The one unit of POD activity was defined as 0.1 increase of absorbance after 1 min per gram of FW.

Guaiacol peroxidase activity (GOPX) was measured according to Chance and Maehly (1955). The supernatant (0.005 ml) was mixed with an activity reagent consisting of 5000 μM guaiacol and 2500 μM hydrogen peroxide in 0.05 M acetic buffer, pH 5.6. Measurements were conducted at 37 °C in Nunc U-bottom 96-well plate on a Varioskan LUX Multimode Microplate Reader. The absorbance at 470 nm was recorded for 10 min with reads every 1 min. The GOPX activity was expressed in μmol of formed tetraguaiacol (ɛ = 26.6 mM^−1^ cm^−1^) per minute and gram of FW.

Ascorbate peroxidase activity (APX) was measured according to Nakano and Asada (1981). The supernatant (0.005 ml) was mixed with an activity reagent consisting of 0.05 M Tris-HCl buffer, pH 7.2, 2000 μM ASA, 5000 μM EDTA and 100 μM hydrogen peroxide. The APX activity was measured at 25 °C in UV-Star 96-well plate on a Varioskan LUX Multimode Microplate Reader by monitoring the rate of ASA oxidation for 10 min with absorbance reads every 1 min at 290 nm. The APX activity was expressed in μmol of ASA breakdown (ɛ = 2.8 mM^−1^ cm^−1^) per minute and gram of FW.

Dehydroascorbate reductase (DHAR) activity was measured according to Trümper et al. (1994). The supernatant (0.01 ml) was mixed with an activity reagent consisting of 0.05 M Tris-HCl buffer, pH 7.2, 4000 μM GSH and 1000 μM dehydroascorbic acid (DHA). The DHAR activity was measured at 30 °C in UV-Star 96-well plate on a Varioskan LUX Multimode Microplate Reader by monitoring the rate of DHA reduction for 10 min with absorbance reads every 1 min at 265 nm. The DHAR activity was expressed in μmol of ASA formation (ɛ = 14 mM^−1^ cm^−1^) per minute and gram of FW.

Glutathione reductase (GR) activity was measured according to Foyer and Halliwell (1976). The supernatant (0.01 ml) was mixed with an activity reagent consisting of 0.05 M Tris-HCl buffer, pH 7.2, 0.25 mM nicotinamide adenine dinucleotide phosphate (NADPH), 1000 μM EDTA and 1000 μM oxidized glutathione (GSSG). Measurements were carried out at 37 °C in Nunc U-bottom 96-well plate on a Varioskan LUX Multimode Microplate Reader and the change in absorbance at 340 nm was monitored for 20 min with reads every 1 min. GR activity was expressed as μmol of oxidized NADPH per minute and gram of FW.

Nitrosoglutathione reductase (GSNOR) activity was measured according to Sakamoto et al. (2002). The supernatant (0.01 ml) was mixed with an activity reagent consisting of 0.05 M Tris-HCl buffer, pH 7.2, 0.2 mM nicotinamide adenine dinucleotide (NADH), 0.5 mM EDTA and 0.6 mM *S*-nitrosoglutathione (GSNO). Measurements were performed at 37 °C in Nunc U-bottom 96-well plate on a Varioskan LUX Multimode Microplate Reader and change in absorbance at 340 nm was monitored for 20 min with reads every 1 min. GSNOR activity was expressed as μmol of oxidized NADH per minute and gram of FW.

Arginase (ARG) activity in supernatants was measured according to Labudda et al. (2016a). Briefly, after the activation of ARG (5 mM MnCl_2_, 56 °C, 10 min), the supernatants were incubated at 37 °C with 0.25 M L-arginine (pH 9.6). The reactions were stopped after 1 h by pipetting the mixture consisting of H_2_SO_4_:H_3_PO_4_:H_2_O (1:3:7, *v*/v/v). Next, 9% α-isonitrosopropiophenone in 96% ethanol was added, and samples were kept at 96 °C for 45 min. Following this incubation, samples were incubated for 10 min in the dark at room temperature. The absorbance was measured at λ = 550 nm in Nunc U-bottom 96-well plate on a Varioskan LUX Multimode Microplate Reader. Urea level was calculated based on the standard curve and ARG activity was expressed as μmol of formed urea per hour and gram of FW.

#### Phenolic metabolites

Leaf samples (100 mg) were ground in a mortar with quartz sand on ice-bath. Phenolic metabolites were extracted from leaves with 5 ml of ice-cold 80% methanol and obtained homogenates were centrifuged for 15 min at 4 °C (16,000×g). The level of total phenols, hydroxycinnamoyl tartaric acid esters, flavonols, and anthocyanins was measured according to Fukumoto and Mazza (2000). The methanol extracts were mixed with 0.1% hydrochloric acid solution prepared in 96% ethanol and 2% hydrochloric acid solution prepared in milli-Q water. Then 15 min after sample incubation in the dark, the absorbance was read in UV-Star 96-well plate on a Varioskan LUX Multimode Microplate Reader. The absorbance at 280, 320, 360, and 520 nm reflected total phenol, hydroxycinnamoyl tartaric acid ester, flavonol, and anthocyanin contents, respectively. The chlorogenic acid (total phenols), caffeic acid (hydroxycinnamoyl tartaric acid esters), quercetin (flavonols), and cyanidin (anthocyanins) were used as equivalents for measurement of specific phenolic metabolites. To estimate the polyphenol content, the Folin-Ciocalteu method was implemented (Labudda et al. 2016b). Concisely, 20 μl of methanol extract was mixed with 1.58 ml of milli-Q water and 100 μl of Folin-Ciocalteu reagent (POCH, Gliwice, Poland). Samples were incubated at room temperature for 4 min, and 300 μl of 1 M saturated sodium carbonate was added and incubation at 40 °C for 30 min was performed. The absorbance was read at 740 nm in Nunc U-bottom 96-well plate on a Varioskan LUX Multimode Microplate Reader and polyphenol content was quantified as gallic acid equivalent. The results of the phenolic metabolite levels were expressed in mg of the respective equivalents per 100 g of FW.

The total salicylic acid (free and conjugated forms of SA) amounts were quantified using the reversed phase high-performance liquid chromatography (RP-HPLC) with fluorescence detection according to Szkop et al. (2017) with small modifications. Briefly, leaf samples (100 mg) of barley plants were homogenized in a mortar with quartz sand and 1.5 ml 0.4 M K_2_HPO_4_, they were vigorously agitated for 15 min at 70 °C and centrifuged at 16,000×g for 10 min. Next, 1 ml of collected supernatants was mixed with 0.15 ml of 10 M HCl and the acidic hydrolysis at 95 °C for 90 min was performed. Afterwards, aliquots (0.85 ml) of ethyl acetate were added to the acidic hydrolysates and samples were vigorously vortexed and centrifuged at 16,000×g for 10 min. The upper organic phases (0.6 ml) were mixed with 200 mM phosphate buffer (pH 7.8) and samples were vigorously vortexed and centrifuged at 16,000×g for 10 min. The lower aqueous phases (0.2 ml) were collected, clarified using 0.45 μm Millex-HV filters (Merck Millipore Ltd., Cork, Ireland) and placed in HPLC vials. The conditions of the HPLC analysis were described in detail in Szkop et al. (2017), and assays were performed using system consisted of a binary pump (Model 1525, Waters Corporation, Milford, MA, USA), a fluorometric detector (Model 474, Waters Corporation) and an autosampler (Model 717plus, Waters Corporation). Chromatographic separations were conducted at room temperature on a C18 column (Symmetry 4.6 × 150 mm, 5 μm, Waters Corporation) guarded by a C18 precolumn (Symmetry 3.9 × 20 mm, 5 μm, Waters Corporation) with a linear gradient elution. The content of SA was quantified based on the external standard calibration curve prepared with the use of HPLC-grade SA (Sigma-Aldrich, Saint Louis, MO, USA).

To visualization of the secondary metabolites, five barley leaves for each treatment at the same developmental stage were taken from randomly chosen plants. Handmade cross-sections were obtained from the middle part of leaf blades by cutting them with a razor blade. The observations were performed in water according to Muszyńska et al. (2019b) under UV irradiation. The fluorescence microscope equipped with a U-MNU narrow-band filter cube (Olympus-Provis, Tokyo, Japan) was used for the autofluorescence detection of the secondary metabolites accumulated in barley leaves.

#### Oxidative damage reflected in lipid peroxidation and protein carbonylation

The 2-thiobarbituric acid reactive substances (TBARs) assay according to Hodges et al. (1999) was implemented. Two hundred μl of methanol extract (obtained as described in Phenolic metabolites paragraph) was added to 800 μl 0.5% 2-thiobarbituric acid dissolved in 20% trichloroacetic acid solution. Then sample incubation at 90 °C for 20 min was performed and reactions were stopped on ice bath. Samples were centrifuged (10 min, 16,000×g) and the absorbance of supernatants was measured at 440, 532 and 600 nm in Nunc U-bottom 96-well plate on a Varioskan LUX Multimode Microplate Reader. TBARs level was calculated and expressed in μmol per gram of FW.

To determine protein carbonylation (carbonyl groups, C=O) level, leaf sample (100 mg) was ground with liquid nitrogen in mortar and 2 ml of extraction buffer (0.1 M phosphate buffer pH 7.2 with 1 mM EDTA, and 0.1% Triton X-100) was added. Homogenate was centrifuged (16,000×g) for 15 min at 4 °C. Supernatant was collected and protein content was measured using Bradford reagent and bovine serum albumin (BSA) (Sigma-Aldrich) as a standard. Extract volume containing 180 μg of proteins was collected, and proteins were precipitated with cold acetone. Protein carbonylation was determined by derivatization of protein carbonyls with 2,4-dinitrophenylhydrazine (DNPH) using a procedure based on Levine et al. (1994). Briefly, 300 μl of 10 mM DNPH in 2.5 M HCl was added to precipitated proteins and incubated for 1 h in darkness with mixing every 15 min. Afterwards proteins were washed 3 times with cold ethanol/ethyl acetate mixture (1:1). Dinitrophenyl group (DNP)-labelled proteins were then dissolved in 90 μl of rehydration buffer containing 7 M urea, 2 M thiourea, 4% 3-[(3-cholamidopropyl)dimethylammonio]-1-propanesulfonate and 40 mM 1,4-dithiothreitol.

Extracts containing 5 μg of derivatized proteins were mixed (1:1, *v*/v) with a sample buffer containing 126 mM Tris-HCl (pH 6.8), 20% glycerol, 4% sodium dodecyl sulphate (SDS), 10% 2-mercaptoethanol and 0.004% bromophenol blue. After incubation at 95 °C for 5 min, protein samples were centrifuged (5 min, 16,000×g) and separated on 11% acrylamide gel with SDS in a 25 mM Tris, 192 mM glycine and 0.1% SDS running buffer (pH 8.3) at 60 V for 15 min followed by 1 h at a constant current of 25 mA per gel until the blue dye front reached the bottom of the gel (Mini-Protean electrophoresis system; Bio-Rad, Hercules, CA, USA). SDS-polyacrylamide gel electrophoresis separated proteins were transferred electrophoretically to a nitrocellulose membrane (Mini-Protean electrophoresis system; Bio-Rad) to detect carbonylated protein levels. After 1 h of blocking at room temperature with 5% non-fat dried milk, the membrane was incubated with anti-DNP polyclonal rabbit antibodies (Sigma-Aldrich; 1:1500) in 10 ml of phosphate-buffered saline (PBS) (pH 7.4) with 0.5% Tween 20. Alkaline phosphatase-conjugated goat antibodies against rabbit IgG (Sigma-Aldrich; 1:20000) were used as the secondary antibodies. The blots were visualized with a standard NBT/5-bromo-4-chloro-3′-indolyphosphate (BCIP) solution containing 0.015% of BCIP and 0.03% of NBT in 10 mL of 0.1 M Tris-HCl buffer, pH 9.5, with 0.1 M NaCl, and 0.05 M MgCl_2_. To determine molecular weight (MW) of proteins, SpectraTM Multicolor Broad Range Protein Marker (Thermo Scientific) was used. Blots were digitalized with G:BOX EF2 (Syngene, Cambridge, UK) and the intensity of bands was quantified as % volume with free BioVision software (Vilber, Collégien, France). The average intensity of all bands was determined. Results were compared with control barley plants, to which a value of 100% has been assigned.

### Statistical analysis

Representative data were shown as means ± SD. Results were subjected to one-way analysis of variance (ANOVA). The significant differences between experimental groups were determined using Tukey’s honest significant difference test at *p* < 0.05. Statistical analysis was performed with Statistica program, version 13.3 (TIBCO Software Inc., Palo Alto, CA, USA).

## Results

**Fig. 1:**
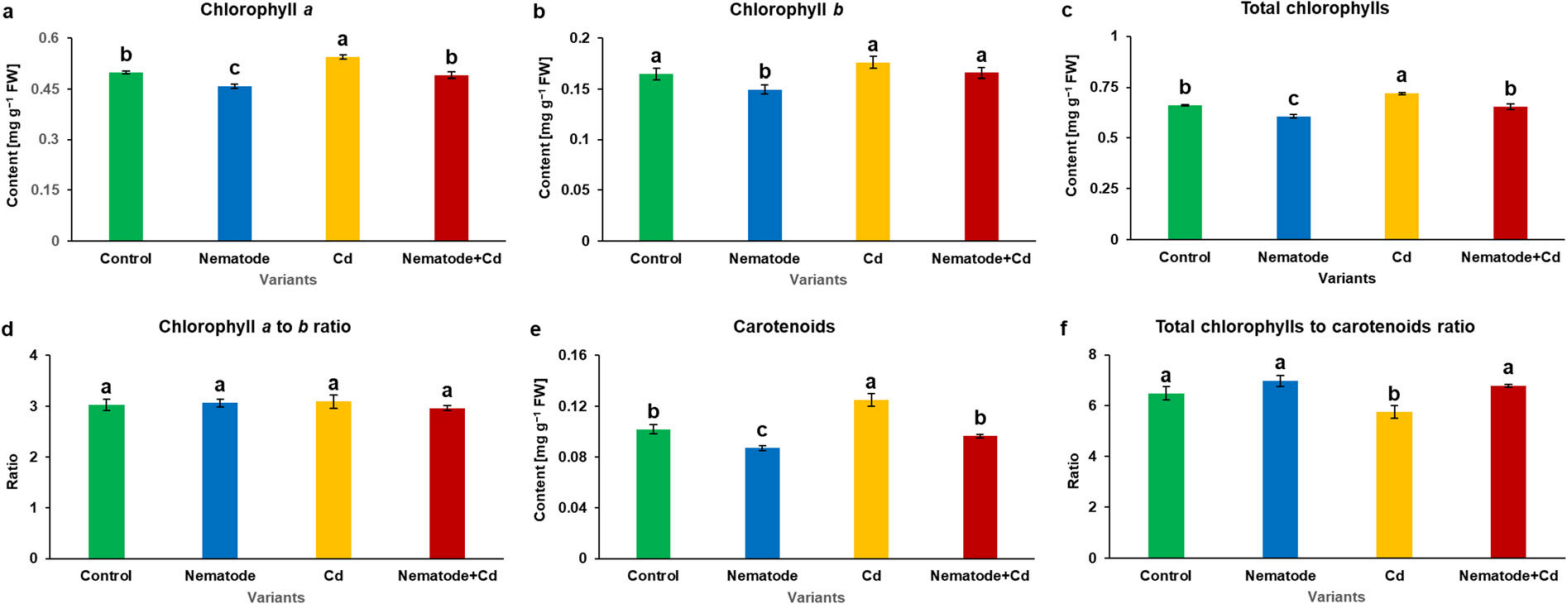
Photosynthetic pigment contents and their ratios (a-f) in the leaves of *Hordeum vulgare* plants cultivated for two weeks on commercial horticultural substrate after *Heterodera filipjevi* inoculation and cadmium application. Results are shown as means ± SD. Different letters indicate means which are significantly different at *p* < 0.05 according to one-way analysis of variance and a post-hoc Tukey’s test

The amount of photosynthetic pigments in leaves of various experimental treatments was differed. Although these values were statistically significant, the changes were not profound (Fig. 1). The content of chl *a* was 0.9-fold lower in N than in C plants and 1.1-fold higher in Cd than in C plants. N + Cd plants presented 1.1-fold higher level of chl *a* in comparison with N plants and 0.9-fold lower than in Cd plants (Fig. 1a). The amount of chl *b* was 0.9-fold lower in N than in C plants (Fig. 1b). The level of total chlorophylls was 0.9-fold lower in N than in C plants and 1.1-fold higher in Cd than in C plants. N + Cd plants showed 1.1-fold higher level of total chlorophylls in comparison with N plants and 0.9-fold lower than in Cd plants (Fig. 1c). It was noticed that values of chl *a*:*b* ratio were on the same level (about 3) in all experimental groups (Fig. 1d). The number of carotenoids was 0.9-fold lower in N than in C plants and 1.2-fold higher in Cd than in C plants. N + Cd plants had 1.1-fold higher amount of carotenoids in comparison with N plants and 0.8-fold lower level as against Cd plants (Fig. 1e). It was observed that the ratio of chl *a* + *b* to carotenoids was 0.9-fold lower in Cd than in C plants and 1.2-fold higher in N + Cd than in Cd plants (Fig. 1f).

**Fig. 2:**
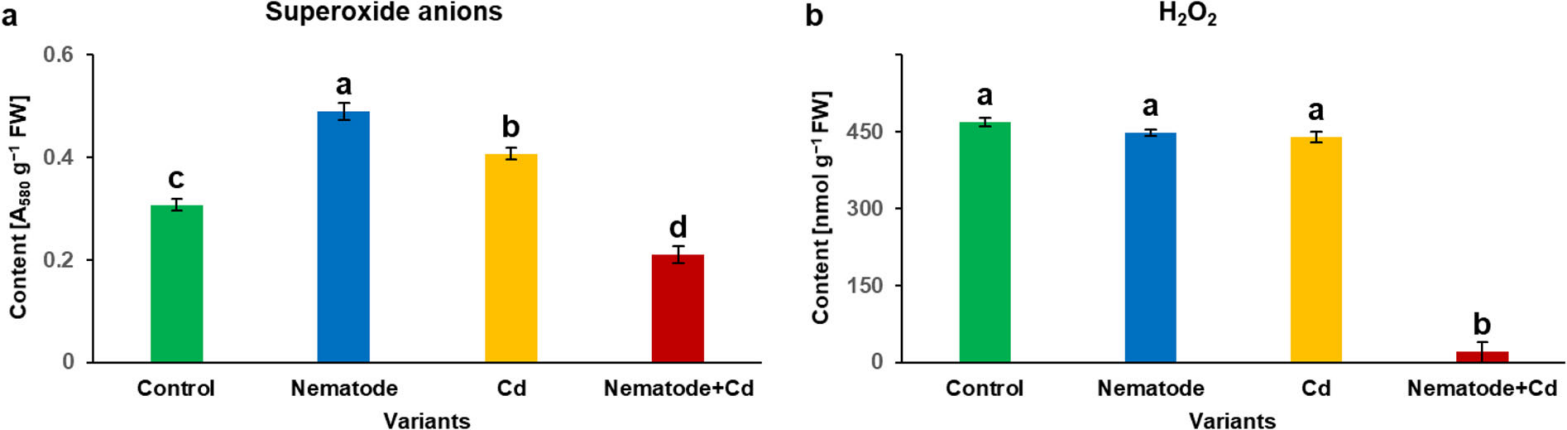
The contents of superoxide anions (**a**) and hydrogen peroxide (H_2_O_2_) (b) in the leaves of *Hordeum vulgare* plants cultivated for two weeks on commercial horticultural substrate after *Heterodera filipjevi* inoculation and cadmium application. Results are shown as means ± SD. Different letters indicate means which are significantly different at p < 0.05 according to one-way analysis of variance and a post-hoc Tukey’s test

The level of superoxide anions was 1.6-fold higher in N than in C plants and 1.3-fold higher in Cd than in C plants. 0.4-fold lower content of superoxide anions was detected in N + Cd plants as against N plants and 0.5-fold lower its content in N + Cd plants than in Cd ones (Fig. 2a). The level of H_2_O_2_ molecules was similar in C, N and Cd plants and it was around 453 nmol g^−1^ (Fig. 2b). Significant decrease in H_2_O_2_ content to about 20 nmol g^−1^ was noted in N + Cd plants in relation to C, N and Cd ones (Fig. 2b).

**Fig. 3:**
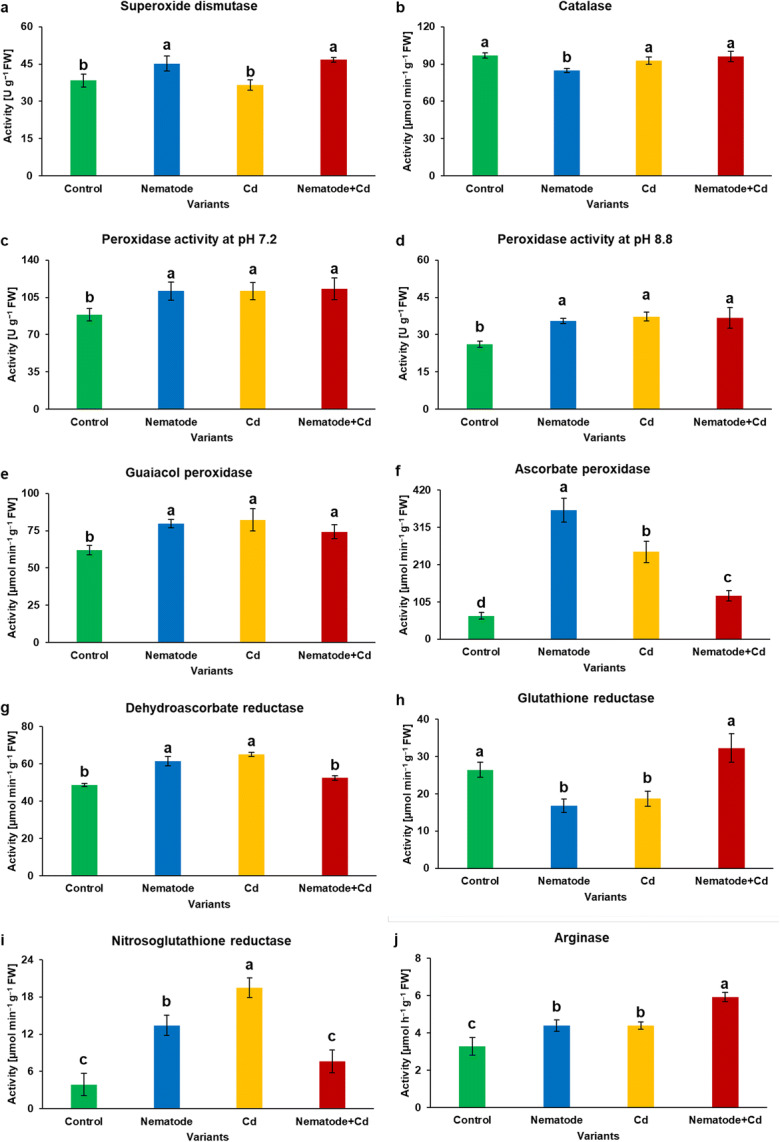
The activity of enzymes (**a-j**) in the leaves of *Hordeum vulgare* plants cultivated for two weeks on commercial horticultural substrate after *Heterodera filipjevi* inoculation and cadmium application. Results are shown as means ± SD. Different letters indicate means which are significantly different at p < 0.05 according to one-way analysis of variance and a post-hoc Tukey’s test

The SOD activity was found to be 1.2-fold higher in N and N + Cd than in C plants and 1.3-fold higher in N + Cd plants in comparison with Cd plants (Fig. 3a). The activity of CAT was down-regulated by 0.9-fold in N plants in relation to controls and up-regulated by 1.1-fold in N + Cd plants in relation to N plants (Fig. 3b). The POD activity showed the same trends at two different pH values, i.e. its activity was stimulated similarly by about 1.3-fold and 1.4-fold at pH 7.2 (Fig. 3c) and pH 8.8 (Fig. 3d), respectively in N, Cd and N + Cd plants as against control plants. The same regularity was also clearly visible in case of GOPX activity as the up-regulation by about 1.3-fold was noted in N, Cd and N + Cd plants when they were compared to C plants (Fig. 3e). Regarding the APX activity, it was found the increase of activity in N, Cd and N + Cd plants by 5.5-, 3.7- and 1.9-folds, respectively in comparison to control plants and APX activity dropped significantly by a third and half in N + Cd plants as against N and Cd plants, respectively (Fig. 3f). As Fig. 3G shows, the activity of DHAR was increased about 1.3-fold in N and Cd plants than in controls, and it was decreased about 0.8-fold in N + Cd plants in comparison both with N and Cd plants. The GR activity presented similar activity level (around 18 μmol min^−1^ g^−1^) in N and Cd plants and this value was lower than activity in C plants. However, the GR activity was meaningfully enhanced in N + Cd plants (about 1.8-fold) in comparison with N and Cd plants (Fig. 3h). In leaves of barley plants separately infected with the cyst nematode and treated with Cd the activity of the GSNOR was significantly stimulated by 3-fold upon infection and 5-fold during the Cd exposition in relation to the control plants, whereas in N + Cd plants statistically significant decrease in its activity was observed. In turn, N + Cd plants did not differ significantly from control plants (Fig. 3i). In the case of ARG, the highest activity was detected in N + Cd plants (about 6 μmol h^−1^ g^−1^) and the lowest (about 3 μmol h^−1^ g^−1^) in control plants. In comparison with C plants, N and Cd plants were characterized by around 1.3-fold stimulated ARG activity (Fig. 3j).

**Fig. 4:**
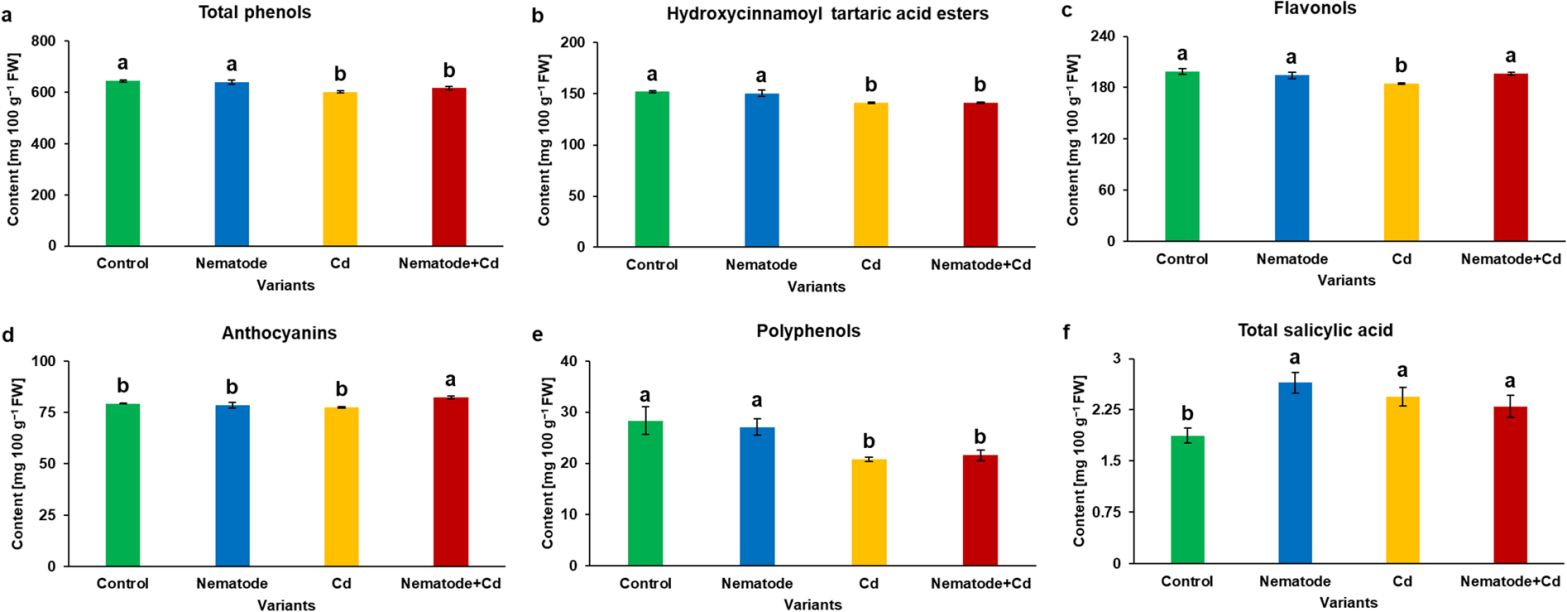
The contents of phenolic metabolites (**a-f**) in the leaves of *Hordeum vulgare* plants cultivated for two weeks on commercial horticultural substrate after *Heterodera filipjevi* inoculation and cadmium application. Results are shown as the means ± SD. Different letters indicate means which are significantly different at p < 0.05 according to one-way analysis of variance and a post-hoc Tukey’s test

Although the accumulation of total phenols, hydroxycinnamoyl tartaric acid esters, flavonols and anthocyanins (Fig. 4a-d) showed statistically significant changes, these parameters gave the impression of being not key influential on plant antioxidant responses during applied stress conditions. However, some trends were noticeable. The above-mentioned parameters did not always differ significantly between N and C plants (Fig. 4a-d). The content of total phenols, hydroxycinnamoyl tartaric acid esters and flavonols decreased by about 7% in Cd plants in comparison with the control ones (Fig. 4a-c). Furthermore, the level of flavonols and anthocyanins was found to be slightly enhanced by about 6% in N + Cd plants as against Cd ones (Fig. 4c,d). Next, it was found that the polyphenol content was significantly decreased by about 26% in Cd and N + Cd plants in comparison with C ones, and its accumulation was diminished by 20% in N + Cd in relation to N plants (Fig. 4e). The total salicylic acid content was about 1.3-fold higher in N, Cd and N + Cd plants than in C plants, while there were no statistically significant differences in SA between N+ Cd plants and N, Cd ones (Fig. 4f).

**Fig. 5:**
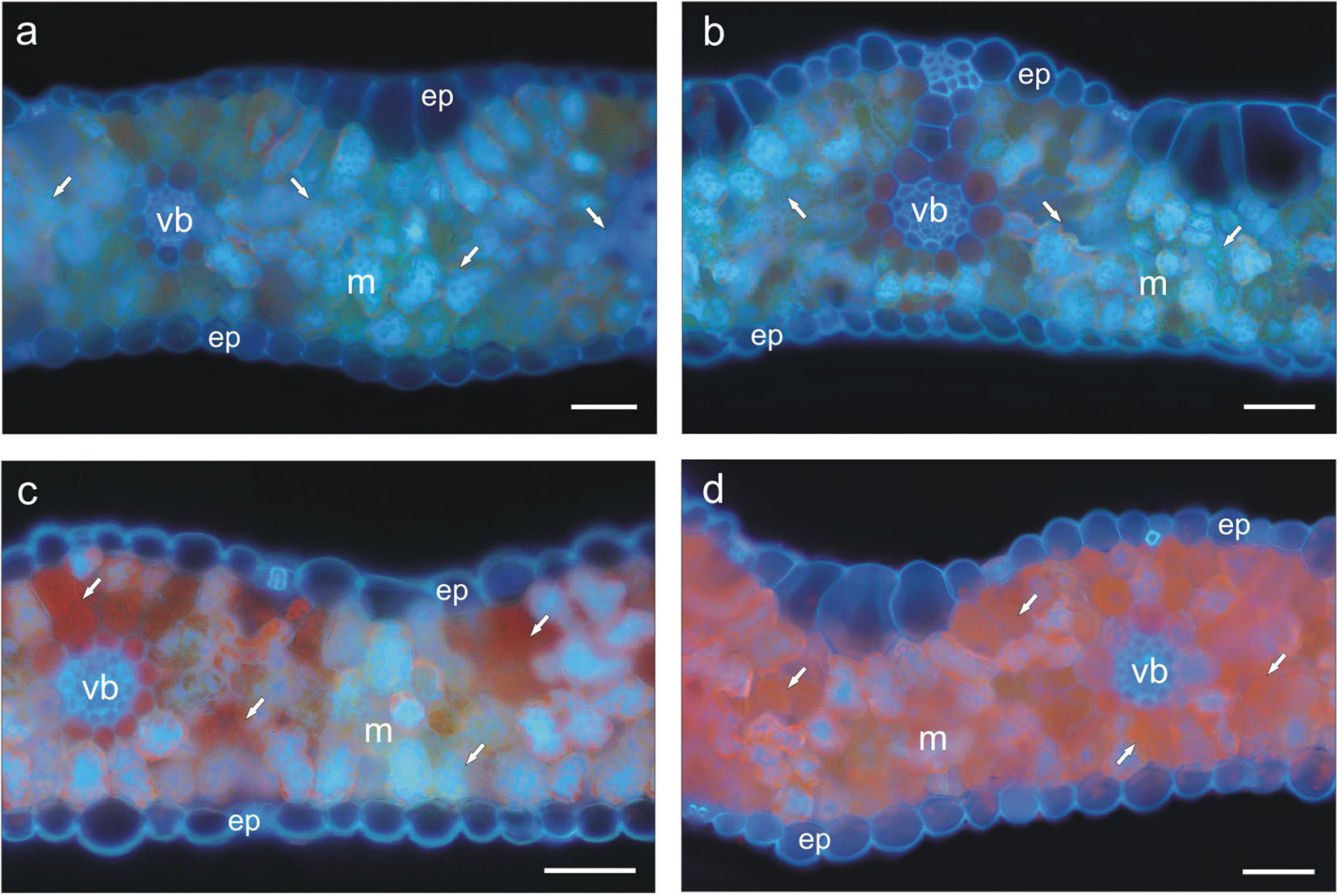
Autofluorescence of secondary metabolites in cross-sectioned leaves of *Hordeum vulgare* nematode-uninoculated and cadmium-untreated (**a**), nematode-inoculated (**b**), cadmium-treated (**c**) and both nematode-inoculated and cadmium-treated (**d**) plants. Bars = 50 μm. Abbreviations: ep, epidermis; m, mesophyll; vb, vascular bundle. Arrows show specific autofluorescence of secondary metabolites

Visualization of secondary metabolites by microscopic method revealed diversification of tested plants (Fig. 5a-d). Upon excitation with UV-light, leaf cells emitted an intensive blue autofluorescence which was brighter in C (Fig. 5a) and N (Fig. 5b) plants than in Cd-treated ones (Fig. 5c). Additionally, in Cd plants red autofluorescence zones appeared (Fig. 5c). Interestingly, when double stress was applied, blue autofluorescence was almost not visible, but N + Cd treatment induced orange-red autofluorescence of mesophyll cells (Fig. 5d).

**Fig. 6:**
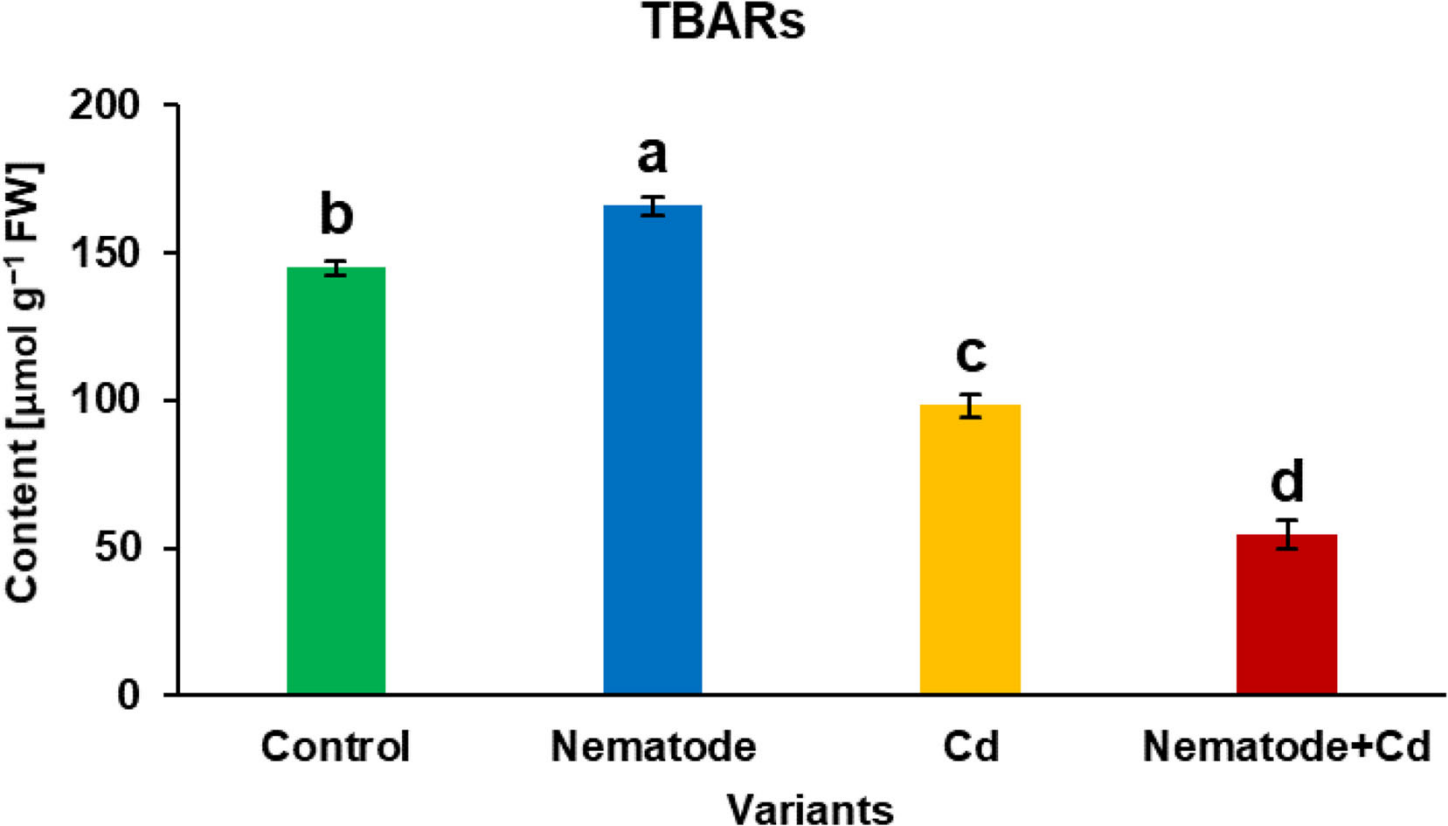
The amount of 2-thiobarbituric acid reactive substances (TBARs), markers of lipid oxidation in the leaves of *Hordeum vulgare* plants cultivated for two weeks on commercial horticultural substrate after *Heterodera filipjevi* inoculation and cadmium application. Results are shown as means ± SD. Different letters indicate means which are significantly different at *p* < 0.05 according to one-way analysis of variance and a post-hoc Tukey’s test

One of the ways to evaluate oxidative damage in plants is measurement of the TBARs content, which is well-known marker of intensity of the lipid peroxidation processes. TBARs level was 1.1-fold increased in N, 0.7- and 0.4-fold decreased in C and N + Cd plants, respectively in comparison with C plants. Moreover, the amount of TBARs was 0.3-fold lower in N + Cd than in N plants and 0.6-fold lower in N + Cd than in Cd plants (Fig. 6).

**Fig. 7:**
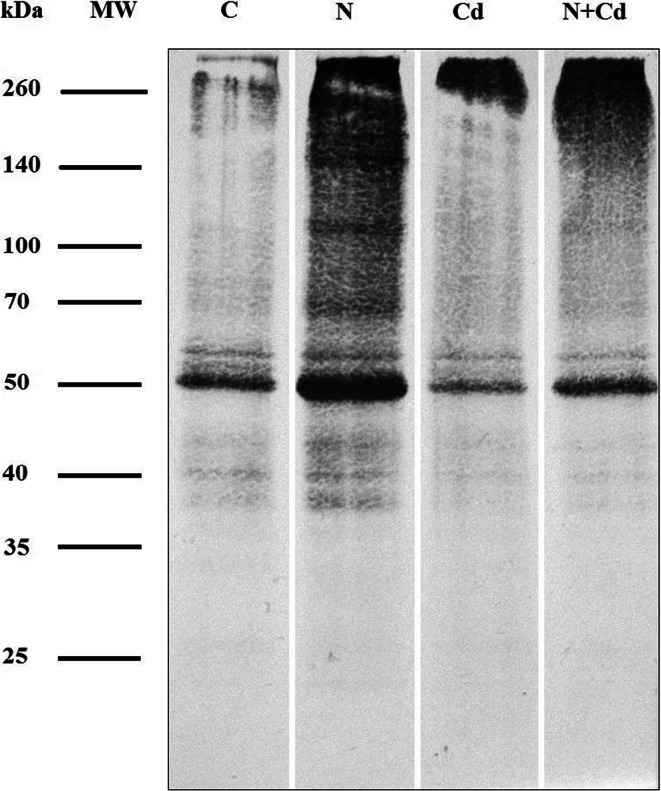
Patterns of carbonylated proteins in the leaves of *Hordeum vulgare* plants cultivated for two weeks on commercial horticultural substrate after *Heterodera filipjevi* inoculation and cadmium application. Abbreviations in order of their occurrence: kDa, kiloDaltons; MW, molecular weight; C, nematode-uninoculated and cadmium-untreated controls; N, nematode-inoculated; Cd, cadmium-treated, N + Cd, both nematode-inoculated and cadmium-treated plants

In addition to the lipid peroxidation, the protein carbonylation level is another marker of intensity of the oxidative damage in plants. As Fig. 7 shows, the general amount of the carbonylated proteins increased by over 76% in barley inoculated with the cyst nematode in comparison with control group of plants. However, in response to Cd, the intensity of protein carbonylation was lowered by 51%, and it dropped by 18% in response to both stresses. The amount of the C=O groups was 1.3-fold higher in N + Cd than in Cd plants and 0.5-fold lower in N + Cd than in N plants (Fig. 7). Not only the overall protein carbonylation, but also patterns of carbonylated proteins were changed. Bands with MW of ~160 kDa, 140 kDa were observed only in N plants. Moreover, in N plants bands with MW of ~110 kDa, and 70 kDa were clearly visible, but they were not detected in Cd plants, and were much less intense in C and N + Cd barley plants. Band with molecular mass of ~58 kDa showed similar intensity in all plant groups. In C plants band with MW ~55 kDa was present, but it was not detected in N + Cd and was much less intense in both Cd and N plants. Carbonylated protein with MW of 50 kDa was also common for all plant groups, however it was the most abundant in N plants, medium intensity was observed in N + Cd as well as in C barley plants, and lowest carbonylation level was noticed in Cd treated plants. Three bands with masses ~45 kDa, 40 kDa 35 kDa were present in all plant groups, but they were the most intense in N and C plants (Fig. 7).

## Discussion

Maintenance of redox balance in plant cells seems to be a universal link connecting plant response to various environmental stresses (Suzuki et al. 2014; Choudhury et al. 2017; Woźniak et al. 2019). To protect cells against oxidative damage, plants evolved several defence mechanisms based on both low-molecular antioxidant compounds as well as on enzymes that directly or indirectly help survive in adverse environmental conditions. Here, we focused on the elucidation of antioxidant mechanisms of spring barley plants during response to abiotic (cadmium exposure) and biotic (cyst nematode infection) stresses occurring simultaneously.

The concentration and composition of photosynthetic pigments are strong prognostic markers of the plant vitality (Blackburn 2007). Our studies showed that the infection with *H. filipjevi* and the treatment with realistic Cd concentration (5 μM) induced only mild changes in pigment parameters in leaves of barley plants. This was because the parasitizing *H. filipjevi* had to guarantee a steady influx of photoassimilates to the syncytial feeding sites in roots from which nematodes drew nutrients for own use. Thusly, despite the stress caused by infestation and cadmium presence, infected plants were maintained alive with an effective photosynthetic apparatus. This was well reflected in the practically constant value of chl *a*:*b* ratio in all four experimental groups. According to Kitajima and Hogan (2003) chl *a*:*b* ratio is an indicator of nitrogen partitioning within leaves and it is positively correlated with the ratio of photosystem II (PSII) cores to light harvesting chlorophyll-protein complex. Thus, despite double stress, the degree of nitrogen nutrition of barley plants remained at a relatively optimal level, so the plants remained still vital (Kitajima and Hogan 2003). The accumulated carotenoids in barley plants that grew in the presence of cadmium are noteworthy. Carotenoids are accessory pigments in chloroplasts. They increase light harvesting and have photoprotective properties, so can quench triplet chlorophyll and singlet oxygen molecules. Singlet oxygen molecules are the main ROS generated during photosynthesis, and their ineffective neutralization can bring photooxidative damage initiated by (a)biotic stresses (Havaux 2014). Accumulated carotenoids in Cd plants could serve as one of the components of the plant non-enzymatic antioxidant machinery and prevent photoinhibition of PSII via non-photochemical quenching and consequently the production of ROS was limited (Zhao et al. 2017).

In the present study, we observed a substantial increase in superoxide anions in barley leaves because of separately *H. filipjevi* parasitism and Cd treatment. Labudda et al. (2018) showed that superoxide and hydrogen peroxide increased level occurred in shoots of *Arabidopsis thaliana* plants infected with the beet cyst nematode *Heterodera schachtii*. In addition, Khanna et al. (2019) presented enhanced production of these ROS in shoots of *Lycopersicon esculentum* plants infested by root-knot nematode *Meloidogyne incognita*. Furthermore, an abundance of published results showed that Cd stimulated the production of ROS in many plant species (Chmielowska-Bąk et al. 2014). A particularly interesting observation concerned decreased level of superoxide anions and barely detectable hydrogen peroxide quantity in leaves of N + Cd plants in comparison with C, N and Cd plants. In part, reduced superoxide anion content was due to the stimulated SOD activity in N + Cd plants in comparison with C and Cd plants. Our results showed that the ROS amount was decreased in double stressed barley plants. This serendipitous finding indicated efficient antioxidant mechanisms, although it could seem that the plant under double stress had disturbed these mechanisms. Suzuki et al. (2014) and Choudhury et al. (2017) suggested that, ROS metabolism and antioxidant mechanisms demonstrated a unique response during double stress, which was dissimilar than response induced by one stressor operating separately. Thus, the combination of two different stresses (abiotic and biotic) imposed on barley plants an exceptional physiological acclimation and ROS metabolism participated in modulation of plant acclimation to double stress.

Class III plant peroxidases (POD and GOPX) take part in numerous physiological processes, for example in lignin and suberin production, the cell wall components cross-linking, formation of phytoalexins and they oxidize broad spectrum of substrates at the cost of H_2_O_2_ molecules (Almagro et al. 2009). There were observed the same trends in GOPX and POD activities in the three stress situations compared to the control plants. The response of class III peroxidases was non-specific in relation to the analysed stress variants and it proved that in the leaves of barley there was a change in ROS metabolism in response to stresses, what is widely known and well documented in the literature (Hasanuzzaman et al. 2019b). The activity of APX, belonging to class 1 peroxidases, was quite different from class III peroxidases, and the differences between plants from four experimental groups were clearly outlined. APX participates in H_2_O_2_ scavenging via Foyer-Halliwell-Asada pathway engaging reduced form of ascorbate and glutathione as an important non-enzymatic antioxidants and enzymes namely, DHAR, GR and monodehydroascorbate reductase (Labudda 2018). Based on obtained results, it was stated that the H_2_O_2_ neutralizing in the leaves of N plants was largely depended on APX activity. This was in line with our earlier studies showing increased APX activity in shoots of *A. thaliana* plants infected with *H. schachtii* (Labudda et al. 2018) and with studies by Khanna et al. (2019) also showing higher activity of APX in tomato leaves as a result of infestation by *M. incognita*. In addition, increased APX activity in Cd plants relative to the control plants found confirmation in results by Tiryakioglu et al. (2006), that showed significantly enhanced activity of APX in barley under Cd exposure.

However, regarding our research hypothesis about an effective antioxidative system under a twofold induced stress in barley plants, our discovery that the activity of APX was dropped in N + Cd plants in relation to N and Cd plants seemed to be a landmark in our study. It should also be mentioned that SA, a known phenolic plant hormone, interplays with ROS and GSH in stressed plants (Herrera-Vásquez et al. 2015). Durner and Klessig (1995) showed that SA reversible inhibited the APX activity with no effect on GOPX. Therefore, the increased number of SA in N + Cd plants could lead to direct inhibition of APX by SA, what altered the cellular redox state and switched on specific defence response in these plants. Our results suggested that H_2_O_2_ scavenging in N + Cd plants was shifted to the pathway of non-enzymatic H_2_O_2_ neutralization by the direct reaction with ASA. Diminished APX activity promoted a higher ASA pool in the cells, which meant that ASA interacted directly with H_2_O_2_ while getting rid of H_2_O_2_ from the cells (Grinstead 1960). As a result of the reaction of H_2_O_2_ with ASA, DHA was formed (oxidized form of ASA), which became a substrate for DHAR. This system worked very well in N + Cd plants, because the DHAR activity was the similar as observed in the control plants, so the enzymatic recovery of the ASA pool was possible. One more observation confirmed our claim. DHAR uses two molecules of GSH to recover the ASA molecule but at the same time GSSG is produced. We observed that the GR activity was strongly stimulated in N + Cd plants. GR catalysed the transformation of GSSG to GSH, so regenerated GSH molecules were still available and/or they might afresh enter to reaction catalysed by DHAR or participate in other GSH-dependent defence mechanisms (Gullner et al. 2018; Labudda 2018). At least three possible options can be considered here. Firstly, H_2_O_2_ might have been reduced by the direct reaction with GSH with simultaneous production of GSSG (Abedinzadeh et al. 1989). Secondly, uptaken and transported to leaves Cd ions might have been chelated by GSH, and formed Cd(GS)_2_ complexes were presumably sequestrated into the vacuoles for detoxification, thus the Cd-induced oxidative damage was limited (Delalande et al. 2010). And finally, thirdly GSH is known to be a substrate for synthesis of phytochelatins (the oligomeric form of GSH). Phytochelatins could form the phytochelatin-Cd complexes and similar to Cd(GS)_2_ complexes, were sequestrated into the vacuoles (Yen et al. 1999; Ahmad et al. 2019).

Conjointly with ROS, reactive nitrogen species (RNS), including nitric oxide (NO), fulfil fundamental roles as mediators of plant growth and development and, they signal in acclimatization and tolerance to stresses (Turkan 2018). Moreover, NO reversibly binds the -SH group of cysteinyl residues and *S*-nitrosothiols (SNOs) are formed, including GSNO, an intracellular reservoir of NO (Jahnová et al. 2019). The level of GSNO in cells is regulated by GSNOR that catalyses the NADH-dependent reduction of GSNO to GSSG and ammonia (NH_3_) (Corpas and Barroso 2013). According to Kovacs et al. (2016) and Begara-Morales et al. (2019) ROS-induced inhibition of the GSNOR activity led to the increased GSNO accumulation in plant cells what resulted in up-regulated H_2_O_2_ scavenging by the Foyer-Halliwell-Asada pathway. Clark et al. (2000) presented the GSNO-mediated irreversible inhibition of APX. This inhibitory effect was time dependent with about 70% inhibition of the APX activity after a 60-min incubation at room temperature. This additionally supports our described above assumptions regarding the regulation of APX activity in N + Cd plants. Results presented by us here and Kovacs et al. (2016) and Begara-Morales et al. (2019) suggest that the unique improved antioxidant capacity of N + Cd plants to some extent resulted from beneficial effect of accumulated GSNO (by inhibition of GSNOR). What is more, Jahnová et al. (2019) indicated that decreased GSNOR activity accompanied by an enhancement in the GSNO level can express in increased plant immunity against pathogens. Moreover, the enhanced GSNOR activity in N and Cd plants was noted, thus removing of excess amount of NO via GSNO breakdown to GSSG and NH_3_ occurred, and effects of ROS and RNS on functioning of barley under separately cyst nematode infection and cadmium exposition were limited (Corpas and Barroso 2013).

Our previous results showed that changes in plant nitrogen metabolism took place during cyst nematode infection (Labudda et al. 2020a). Arginase (ARG), nitrogen metabolism-linked enzyme, was another enzyme that was analysed in these studies. ARG is not an enzyme that directly participates in the metabolism of ROS, but it supplies substrate for the synthesis e.g. polyamines, important compounds in plant antioxidant response (Hasanuzzaman et al. 2019a). ARG produced ornithine and urea, and next ornithine was decarboxylated by ornithine decarboxylase to putrescine (diamine). Spermidine synthase from putrescine synthesised spermidine (triamine), and spermine synthase converted spermidine to spermine (tetramine). Mentioned above polyamines are positively charged molecules, so they might bind to opposite charged molecules and probably might scavenge ROS (Hasanuzzaman et al. 2019a). Therefore, stimulated ARG activity in N + Cd plants might promote polyamine synthesis and indirectly enhance non-enzymatic antioxidant mechanism.

Antioxidative mechanisms dependent on phenolic compounds did not play a key role in this experimental model. The content of phenolics (although some alternations at a statistically significant level) did not change to such an extent that their holistic physiological impact on plants could be assumed, and thus they were rather proved to only support other antioxidant responses operating in plants. The only thing to notice was the reduced polyphenol content in plants treated with Cd and under the influence of double stress. Polyphenols due to their interaction with ROS/RNS limit oxidative damage in cells (Hussain et al. 2016). The observed decline in polyphenol level in Cd and N + Cd plants resulted probably from their intake during response against these stress conditions. Spectrophotometric measurements of phenolic compounds were in accordance with microscopic analysis, which revealed lower intensity of blue autofluorescence of vacuolar compounds in Cd and N + Cd plants than in other treatments. It is also noteworthy that these two groups of plants were characterized by red autofluorescence of anthocyanins which was more visible in N + Cd leaves than in Cd ones. This statement was also confirmed by enhanced level of these compounds’ accumulation in double-stressed plants.

In our studies, we used two biochemical markers for the estimation of the oxidative damage intensity. Firstly, TBARs assay for the polyunsaturated fatty acids oxidation products detection, such as aldehydes, alkenals and hydroxyalkenals including particularly toxic molecules, 4-hydroxy-2-nonenal and malondialdehyde, was used (de Dios Alché 2019). Secondly, the western blotting profiling for the C=O groups formed in proteins as a result of the amino acid residues (mainly Arg, Pro, Thr and Lys) oxidation by ROS (protein carbonylation) was implemented (Kalemba and Pukacka 2014). It turned out that our hypothesis of higher antioxidant activity in N + Cd plants appeared to be true. The above discussed defence mechanisms working together proved to be efficient enough to prevent excessive lipid and protein oxidation by ROS. The ROS level was after all also reduced in these plants. Interestingly, the content of TBARs and the C=O groups was even lower in N + Cd than in control plants, so an integrity of cell membranes of these plants was maintained and cell damage and loss of the protein functions were significantly limited.

## Conclusions

The presented results expand the knowledge of defence mechanisms of barley treated with two contrasting stresses of different origin. For the first time, the involvement of antioxidants in responses against oxidative stress induced jointly by cyst nematodes and cadmium ions was characterized in detail. Our findings uncovered that the ROS neutralization machinery presented a remarkable effectiveness during double stress, which differs from that stimulated by one stressor acting singly. Therefore, simultaneous application of two stressors imposed on barley plants an unusual physiological acclimation expressed in a significant reduction of oxidative damage in double stressed specimens. Summarizing, to manage the acute Cd stress with short exposure time adapted to the dynamics of growth and development of cyst nematode larvae in roots under conditions of our pot experiment, barley plants could rapidly induce a multi-component model of stress response, in order to detoxify Cd ions and efficiently repair damage caused by double stress.

## Abbreviations

APX: Ascorbate peroxidase
ARG: Arginase
ASA: Reduced ascorbate
CAT: Catalase
Chl *a*: Chlorophyll *a*
Chl *b*: Chlorophyll *b*
DHAR: Dehydroascorbate reductase
FW: Fresh weight
GOPX: Guaiacol peroxidase
GR: Glutathione reductase
GSH: Reduced glutathione
GSNO: *S*-Nitrosoglutathione
GSNOR: Nitrosoglutathione reductase
GSSG: Oxidized glutathione
POD: Peroxidase
PSII: Photosystem II
RNS: Reactive nitrogen species
ROS: Reactive oxygen species
SA: Salicylic acid
SNO: *S*-nitrosothiol
SOD: Superoxide dismutase
TBAR: 2-Thiobarbituric acid reactive substance
